# Bone density of the femoral neck following Birmingham hip resurfacing: A 2-year prospective study in 27 hips

**DOI:** 10.3109/17453670903486992

**Published:** 2009-12-04

**Authors:** Nick J Cooke, Lauren Rodgers, David Rawlings, Andrew W McCaskie, James P Holland

**Affiliations:** ^1^North Tees and Hartlepool Hospital; ^2^Statistics Department, University of Newcastle; ^3^Medical Physics; ^4^Orthopaedics, Freeman Hospital, Newcastle upon Tyne, UK

## Abstract

**Background** Resurfacing is a popular alternative to a standard hip replacement in young arthritic patients. Despite bone preservation around the femoral component, there is little information regarding the bone quality.

**Patients and methods** 32 patients underwent consecutive Birmingham hip resurfacing. The bone density of the femoral neck was measured preoperatively and then at 6 weeks, 3 months, 1 year, and 2 years. The femoral neck was divided into regions of interest. Results were available for 27 hips in 26 patients.

**Results** The overall femoral neck bone density showed a trend towards a decrease at 6 weeks and 3 months but returned to the preoperative level at 1 year, and was maintained at 2 years. The combined superior regions of the neck showed a statistically significant decrease in bone density at 6 weeks and 3 months. This returned to preoperative levels at 1 year and was maintained at 2 years.

**Interpretation** Bone density appears to decrease at 6 weeks and 3 months, suggesting that care is necessary until bone density begins to recover.

## Introduction

Resurfacing is emerging as a popular alternative to standard hip replacement in the young, osteoarthritic, active patient (Treacy et al. 2005). In the most recent report from the UK National Joint Registry, resurfacing accounted for 10% of all hip replacements and 50% of hip replacements in patients less than 55 years old. The theoretical advantages of resurfacing include preservation of bone, in particular proximal femoral bone, which aids any revision surgery to a standard hip replacement (Ball et al. 2007). Despite obvious preservation of bone on radiographs, little has been published regarding the quality of this bone postoperatively.

The incidence of femoral neck fracture following resurfacing has varied from 0 to 10% between studies. An Australian series of over 3,000 patients has shown an incidence of 2% in women and 1% in men, with fracture occurring early, on average 15 (13–18) weeks after surgery (Shimmin and Back 2005). The cause of femoral neck fracture is thought to be multifactorial. Studies have shown an association with avascular necrosis (Steffen et al. 2005), varus placement of the femoral component (Shimmin and Back 2005), notching of the femoral neck (Beaule et al. 2006), small femoral heads in male patients, unseated components leaving femoral bone exposed, large cysts within the femoral head, and osteoporosis (Amstutz et al. 2006).

Thinning of the femoral neck after resurfacing has also been reported. The etiology is again unclear but may be related to alteration in bone biology. Neck-thinning was reported to occur in 125 of 163 patients and 45 of them showed a loss of diameter of 10% or more (Hing et al. 2007). The decrease in neck diameter occurred within the first 3 years and then stabilized.

Kishida et al (2004) investigated bone density around the proximal femur in patients who underwent resurfacing and uncemented standard total hip replacement. They concluded that bone density increased in the Gruen zones of the proximal femur and in the femoral neck area, although no preoperative or early postoperative bone density measurements were performed for comparison.

Harty et al. (2005) measured the bone density of the femoral neck after resurfacing and compared the results with those from the contralateral unoperated hip. They concluded that bone density was similar in the operated and unoperated hip, although the bone density measurements were only performed postoperatively with no measurement preoperatively or during the “at risk” period.

Stress shielding would be expected to cause weakening of the bone around and under the implant, and strengthening of the bone distally in the femur. The above studies suggest that the density does not change.

To the best of our knowledge, there have been no longitudinal studies of bone mineral density (BMD) in the femoral neck before and after resurfacing. In this prospective study, we measured the BMD of the femoral neck in patients undergoing hip resurfacing—both preoperatively and postoperatively using dual-energy X-ray absorption (DEXA).

## Patients and methods

The study involved 30 patients (32 hips, 16 men) who consecutively underwent Birmingham hip resurfacing (BHR) (Midland Medical Technologies, Birmingham, UK) over a 9-month period. 25 patients suffered from osteoarthritis and 1 patient had rheumatoid arthritis. The study received ethical approval from the Newcastle and North Tyneside Local Research Ethics Committee (ref. 2002/234) and patients were included after giving their consent.

A single surgeon (JH) performed all the procedures, which were carried out using a posterior approach. A standard operative technique was used in all cases, using the method described by et al. (1996) but without the use of a femoral vent. Components were cast from cobalt chrome. Fixation on the acetabular side was cementless (hydroxyapatite-coated) and on the femoral side it was cemented using a reduced viscosity cement (Simplex; Stryker Howmedica, Allendale, NJ). In each case, antibiotic and DVT prophylaxis was provided—3 doses of cefuroxime, and foot pumps and TED stockings.

A standard postoperative regime was used. All patients were mobilized on day 1 with protected full weight bearing on crutches for between 4 and 6 weeks.

The BMD was measured using DEXA. A zonal reporting technique was employed. This technique has been shown to be reliable and reproducible (Murray et al. 2005) with an intraclass correlation (i.e. the correlation between any 2 assessments of 1 region of interest) of 0.997, with an overall coefficient of variation of 5%. Preoperative DEXA scans were performed in the routine pre-assessment clinic 2 weeks before surgery and then 6 weeks, 3 months, 1 year, and 2 years after surgery.

**Figure 1.**
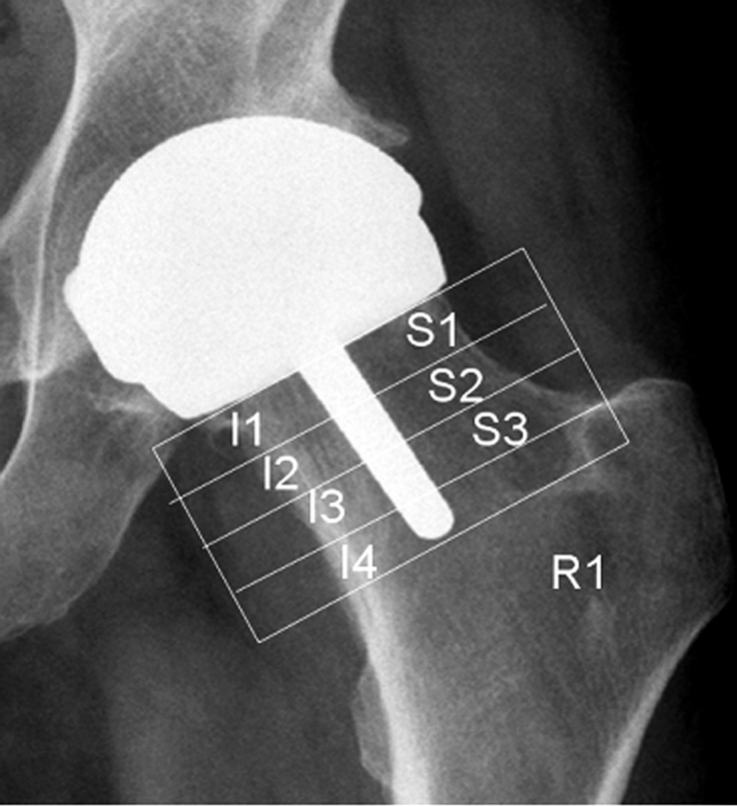
The superior and inferior zones of the femoral neck after Birmingham hip resurfacing.

DEXA was performed using a Hologic QDR 45000A scanner. Each patient was placed supine on the table. The Hologic prosthetic foot positioner was used; it immobilizes the foot and ankle in 0 degrees of internal rotation, which allows a reproducible DEXA scan of the hip. Each scan was analyzed using the Hologic prosthetic scan analysis software (operating system 9.80; v. 8.26a:3). At the 6-week scan, a variable number of equal-sized regions of interest (ROI) in the femoral neck were identified in each patient in relation to the prosthesis (Figure 1). Each region was of constant width, according to the scanning software. Regions were numbered consecutively away from the prosthesis towards the trochanteric line, in order to have comparable periprosthetic regions for analysis of subsequent scans of each patient and for comparisons between patients. The number of regions varied for each patient, depending on the length of the femoral neck, and they were numbered superiorly moving away from the prosthesis (S1, S2, S3 etc.). This was repeated inferiorly away from the prosthesis towards the trochanteric line (I1, I2, I3).

The template for the 6-week scan was then transferred (using the software) to the preoperative, 3-month, 1-year, and 2-year scans. This technique produces identical areas of interest for each scan to allow comparison of results.

BMD was expressed as a percentage of each patient’s original preoperative BMD to show relative changes with the passage of time for each patient. This would allow for individual differences in the absolute variation in BMD between patients due to age, body weight, menopausal status, physical activities, etc.

Alignment of the femoral prosthesis was measured using plain AP and lateral radiographs of the hip with a standard foot positioner. The AP alignment was calculated by measuring the angle between the femoral shaft and the stem of the femoral prosthesis. By dividing the femoral neck into thirds and measuring the position of the tip of the femoral stem within the neck, anterior, central or posterior calculated the lateral alignment.

### Statistics

Repeated measures ANOVA was used to analyze each region; the normality assumption was satisfied in all cases. Pairwise comparisons adjusted with a Bonferroni correction from the ANOVA were used to assess any significant differences between the 4 postoperative scans and the preoperative bone density. The sex of the subject was added as a between-subject factor in the ANOVA. Any p-value of < 0.05 was considered significant. Analysis was done using SPSS version 14.0.

## Results

Results were available for 27 hips in 26 patients (13 women); 4 patients were excluded as they lacked final scan results. After exclusion, the average age of female patients was 50 (27–62) years and for males it was 54 (36–67) years.

Radiographic alignment of the femoral component was measured on standard postoperative plain radiographs with a mean of 139 (127–146) degrees in the AP plane. In the lateral plane, 21 were aligned in the central third of the femoral neck with 6 entering the anterior third of the neck. AP and lateral radiographs did not show any femoral neck thinning at 2 years.

There was 1 case of heterotopic ossification, Brooker grade 1, which appeared not to overlie any regions of interest in the femoral neck for DEXA measurement. No fractures were detected in the study group. There were no other untoward events noted to influence recovery, e.g. thromboembolism or infection. No notches were observed.

### Analysis by regions

**Figure 2.**
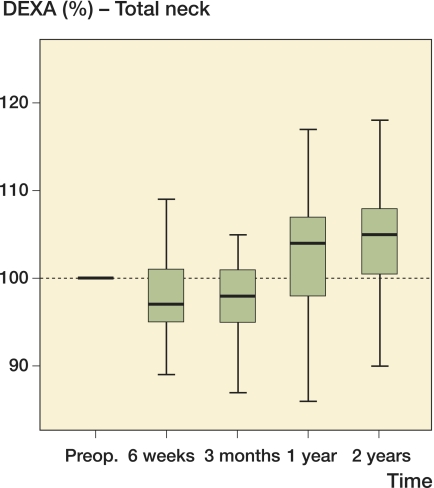
Graph showing percentage change in bone density in the whole of the femoral neck over time, represented as boxplots with mean, inter-quartile range (boxes), and range of values excluding outliers.

Values for overall femoral neck BMD (total of all regions) showed a trend towards a decrease in density at 6 weeks and 3 months and a return to preoperative levels by 1 year. This was maintained at 2 years. The changes were not statistically significant (Figure 2).

**Figure 3.**
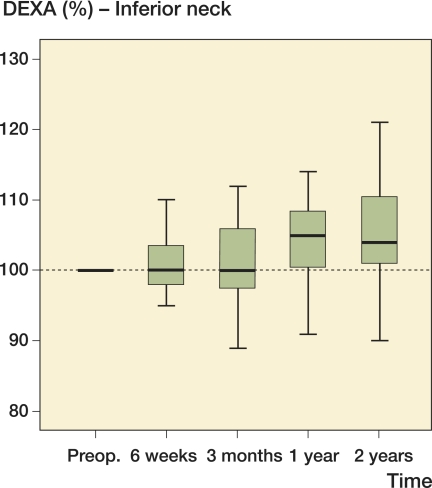
Graph showing percentage change in bone density in the inferior femoral neck over time, represented as box plots with mean, inter-quartile range (boxes), and range of values excluding outliers.

Review of the overall total BMD of the combined inferior regions showed that BMD was maintained, with no significant difference compared to preoperative values, at 6 weeks and 3 months, 1 year, and 2 years (Figure 3).

**Figure 4.**
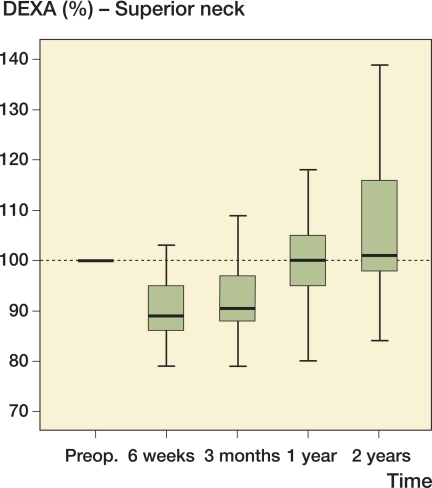
Graph showing percentage change in bone density in the superior femoral neck over time, represented as box plots with mean, inter-quartile range (boxes), and range of values excluding outliers.

Overall total BMD of the superior regions decreased by an average of 9% (p < 0.001). At 3 months, the mean decrease was 8% (p < 0.001) but this recovered to return to preoperative levels by 1 year and was maintained at 2 years (Figure 4).

Looking in more detail at individual zones, at 6 weeks decrease in the mean BMD compared with preoperative levels was seen in superior regions 1 and 2 (9% and 8%, respectively) (p < 0.001 in both cases) At 3 months, the BMD in superior region 1 continued to show a decrease in mean bone density of 8% (p < 0.001), although this subsequently returned to preoperative levels by one year—with maintenance or improvement thereafter.

Inferior region 2 showed a mean decrease in bone density of 7% (p = 0.05) but this returned to normal at 1 year and was unaltered at 2 years. Increased mean BMD was found at 6 weeks in inferior region 3 (p = 0.006) and inferior region 4 (p = 0.02) (7% and 9%, respectively). Inferior regions 3 and 4 continued to show a mean increase in BMD at 3 months (of 6% and 10%; p = 0.03 and p = 0.001, respectively), which was maintained at the 1-year and the 2-year scans.

In summary, the largest decrease in mean BMD was seen in the regions immediately adjacent to the femoral component in superior regions S1 (9%) and S2 (8%) and in inferior region I2 (7%). The largest increases were seen in the inferior regions of the neck towards the inter-trochanteric line, with a mean increase of 10%, which was maintained at 2 years.

**Figure 5.**
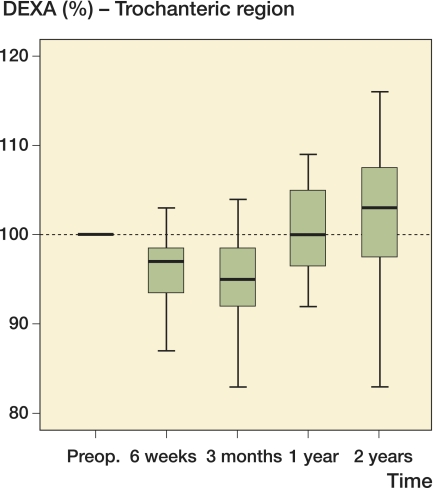
Graph showing percentage change in bone density in the femur lateral to the inter-trochanteric line (R1) over time, represented as box plots with mean, inter-quartile range (boxes), and range of values excluding outliers.

Region R1, the extracapsular trochanteric zone (equivalent to Gruen zones 1 and 7) also showed a decrease in BMD of 4% at 6 weeks (p = 0.002) and of 5% at 3 months (p = 0.001) but BMD returned to preoperative levels at 1 year and was unaltered at 2 years (Figure 5).

No statistically significant changes in BMD of any zone were detected with regard to the sex of the patient.

## Discussion

In this study, we report bone density data that demonstrates preservation and improvement in overall femoral neck bone density following Birmingham hip resulfacing, at 1 and 2 years. However, we also found a reduction in bone density at 6 weeks and 3 months in regions of periprosthetic bone and the superior neck. This returned to preoperative levels by 1 year, and continued to rise at 2 years to exceed preoperative levels.

Our results follow on from the studies by Kishida et al. (2004) and Harty et al. (2004), which showed postoperative increases in BMD. The difference may be explained by the absence of preoperative and early postoperative measurements in these studies, which would have missed any early changes in BMD.

The cause of the reduction in BMD at 6 weeks and 3 months is probably multi-factorial. During surgery, the hip is compromised by capsular release and the subsequent vascular disruption is combined with that of dislocation and torsion of the leg (Kahn et al. 2007) The bony architecture of the head and neck suffers mechanical assault during femoral preparation, followed by the potential effects of thermal injury from cement polymerization and embolic events from implant pressurization and impaction.

Studies of intraoperative oxygen concentration at the femoral head during resurfacing have shown that the blood supply is compromised in all patients, with a mean drop of 60% during the surgical approach and a further 20% during component insertion (Steffen et al. 2005). Histological studies of femoral heads retrieved after resurfacing have shown that most of the bone in the femoral heads is alive (92%), although the bone was found to be dead in 2 cases that had both failed due to fracture (Bradley et al. 1987). Despite these findings, recent studies with PET scanning have shown that the femoral head is viable (Forrest et al. 2006, Mcmahon et al. 2006).

The transient dip in region R1, lateral to the trochanteric line, suggests that an alteration in blood supply may not be the only cause of the transient BMD dip. Region R1 is extracapsular; thus, the blood supply should not be affected by resurfacing, especially as the femoral canal has not been breached.

Changes in mechanical loading of the femur after resurfacing have been hypothesized to be a cause of neck fracture, and may be reflected in change in BMD. A recent study using a computer model of the hip predicted bone resorption in the inferomedial and superolateral bone within the Birmingham resurfacing shell (Ong et al. 2006). This would seem to be reflected by our results, with such resorption being a cause of the initial reduction in density in the zones adjacent to the prosthesis. The improvement in density over time may be due to remodeling. This theory has, however, been disputed in other cadaveric studies that have suggested that bone stresses predicted after resurfacing in both normal and aged femoral neck would not be sufficient to be a possible cause of fracture (Little et al. 2007).

Radcliffe and Taylor (2007) suggested that the cement mantle may be important in altering the strain effects in the femoral neck. A thicker cement mantle increased strain shielding in the superior resurfaced femoral neck (Radcliffe and Taylor 2007). This coincides with the decrease in bone density in the superior region but does not fully explain the recovery in BMD or the time scale of reduction followed by recovery.

Our findings suggest that the maximum decrease in BMD coincides with the most common time of fracture: 13–18 weeks (Shimmin and Back 2005). Women are also thought to be at a higher risk of fracture than men (2% incidence in females and 1% incidence in males (Shimmin and Back 2005) but we found no statistically significant difference in bone density between men and women. The largest drop in density was in a man.

Our study has implications for rehabilitation. To our knowledge, all patients complied with postoperative advice regarding full weight bearing but with use of crutches for balance purposes. Variations in patient compliance to postoperative instructions may have led to minor differences in effects on each individual patient; however, one would expect this to affect the patient’s overall BMD in every zone, not in selected zones. Thus, changes in BMD in certain zones with time in an individual patient should be independent of the weight bearing on that limb.

Our study regime postoperatively consisted of protected full weight bearing with crutches for 4–6 weeks. By 6 weeks, the patient would be fully weight-bearing and commencing non-impact stretching range-of-movement exercises, such as swimming, exercise bike, rowing machine, or cross-trainer. At 6 months gentle jogging was permitted and by 1 year unlimited activity including impact sports, if desired, was the aim.

The period of protected weight bearing should take account of the surgeon’s feel for cup stability and head seating, alignment and clearance of notching, and the patient’s stability, comfort and safety. Most surgeons recommend 6 weeks as standard. In the light of our findings, added care from impact activity would be necessary between 6 weeks and 3 months, especially if known risk factors are present such as notching or varus placement.

No fractures occurred in our study group; therefore, further larger studies are needed to investigate whether bone density is associated with fracture—and if so, whether the risk of fracture could be predicted by a preoperative DEXA scan.

It is important to note that our results should not be extrapolated to other designs of resurfacing. Different internal head geometry, stem design, and cementation techniques may confer different load transfer mechanics to the femoral head and neck. Different metal surfaces and clearance diameters will confer different fluid dynamics, friction, and wear characteristics to the joint and may therefore lead to different changes in bone density.

The temporary reduction in density should be interpreted with caution. It would appear to have been of no clinical relevance in this series, and previous studies of RSA in BHR have shown that the femoral component is perfectly stable during the immediate 2-year postoperative period (Glyn-Jones et al. 2004, Itayem et al. 2005). However, if it is assumed that there is a relationship between BMD and bone strength, this initial decrease does seem to coincide with the reported time of neck fracture in the Australian register, but it may only be clinically significant if combined with other mechanical weakness such as varus femoral component position or notching of the femoral neck.

It will be of interest to follow up these cases in the long term, to establish whether there is any correlation between early changes in BMD and later incidence of neck thinning or avascular necrosis, as A recent study has shown 10% thinning of the neck in 15% of patients at 5 years (Heilpern et al. 2008).
